# Biofilms and Cyclic di-GMP (c-di-GMP) Signaling: Lessons from *Pseudomonas aeruginosa* and Other Bacteria*[Fn FN2]

**DOI:** 10.1074/jbc.R115.711507

**Published:** 2016-04-21

**Authors:** Martina Valentini, Alain Filloux

**Affiliations:** From the MRC Centre for Molecular Microbiology and Infection, Department of Life Sciences, Imperial College London, London SW7 2AZ, United Kingdom

**Keywords:** antibiotic resistance, biofilm, cyclic di-GMP (c-di-GMP), Pseudomonas aeruginosa (P. aeruginosa), signaling

## Abstract

The cyclic di-GMP (c-di-GMP) second messenger represents a signaling system that regulates many bacterial behaviors and is of key importance for driving the lifestyle switch between motile loner cells and biofilm formers. This review provides an up-to-date *compendium* of c-di-GMP pathways connected to biofilm formation, biofilm-associated motilities, and other functionalities in the ubiquitous and opportunistic human pathogen *Pseudomonas aeruginosa*. This bacterium is frequently adopted as a model organism to study bacterial biofilm formation. Importantly, its versatility and adaptation capabilities are linked with a broad range of complex regulatory networks, including a large set of genes involved in c-di-GMP biosynthesis, degradation, and transmission.

## Introduction

Bacteria can live as planktonic cells exploring aqueous environments or as a sessile biofilm community. The switch from planktonic to sessile occurs when, under propitious conditions, individual cells encounter a surface and undergo a series of dramatic physiological, metabolic, and phenotypic changes. Among these changes are the slowdown of metabolic activities and the production of an extracellular matrix, a complex mixture of exopolysaccharides, proteins, and nucleic acids (1). In the case of pathogens, the two bacterial lifestyles also differ in terms of virulence factor production and infection strategies. Although planktonic cells cause fulminant acute infections, the formation of a biofilm correlates with deep-rooted chronic infections and resistance to both phagocytosis and antimicrobial agents (2).

Cyclic di-GMP (c-di-GMP)^3^ is recognized as an intracellular signaling molecule coordinating the “lifestyle transition” from motility to sessility and vice versa (*i.e.* dispersion) (3). The correlation between high c-di-GMP concentration in the cell and biofilm formation or between low c-di-GMP levels and motility has been demonstrated in several bacteria species, *e.g. Escherichia coli*, *Pseudomonas aeruginosa*, and *Salmonella enterica* serovar Typhimurium (4). *P. aeruginosa* biofilms are estimated to contain on average 75–110 pmol of c-di-GMP per mg of total cell extract, whereas planktonic cells contain less than 30 pmol mg^−1^ (5). This concept is widely accepted but does not include the multiplicity of c-di-GMP transmission cascades operating during biofilm. Biofilm determinants modulated by c-di-GMP range from flagella rotation to type IV pili retraction, exopolysaccharide production, surface adhesin expression, antimicrobial resistance and other stress responses, secondary metabolite production, and biofilm dispersion (3). How do we reconcile the global effect of the intracellular c-di-GMP concentration on stimulating the biofilm lifestyle with the discrete actions of c-di-GMP on biofilm formation? Biofilm formation is considered as a developmental process that includes attachment to and movement on the surface, formation of microcolonies, maturation, and ultimately dispersal (1, 6, 7). It is proposed that cells use c-di-GMP as a checkpoint to proceed through the distinct stages of biofilm development until they fully commit to the biofilm lifestyle, although they may still be offered the choice to revert the decision at any time (3, 8).

## The c-di-GMP Metabolism

**FIGURE 1.**
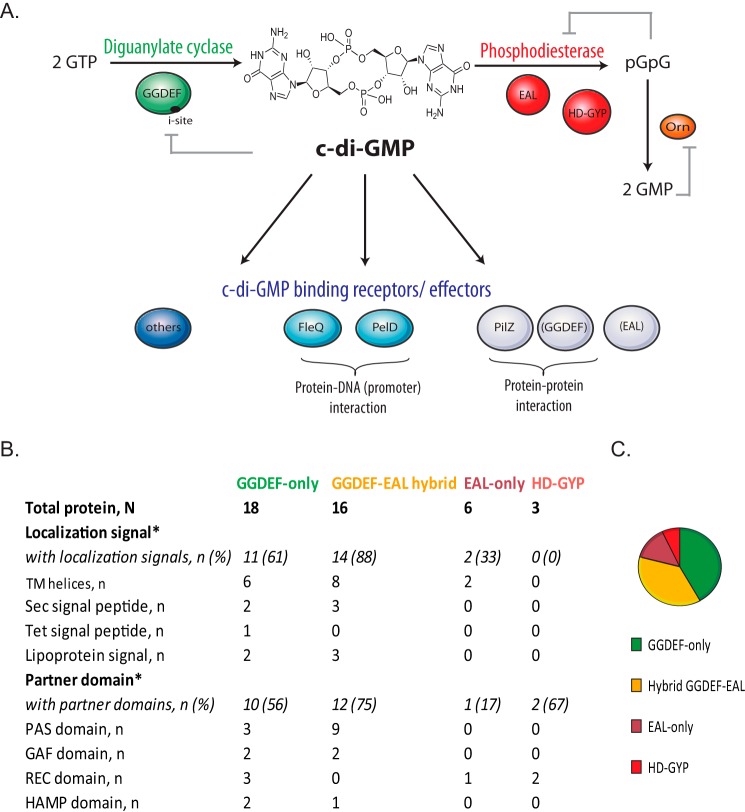
**Molecular basis of c-di-GMP signaling in *P. aeruginosa*.** *A*, c-di-GMP is synthesized by diguanylate cyclases (*green*) that carry GGDEF domains and degraded by phosphodiesterases (*red*) that carry either EAL or HD-GYP domains. EAL phosphodiesterases linearize c-di-GMP into pGpG, which is successively hydrolyzed into 2 GMP molecules primarily by the oligoribonuclease Orn (*orange*) (34, 35). HD-GYP-phosphodiesterases are proposed to perform both steps of the c-di-GMP degradation process (31). Feedback inhibition mechanisms are illustrated by *gray lines*. In the cell, c-di-GMP regulates cellular processes at different levels (transcriptional, post-transcriptional, and post-translational). The diversity of c-di-GMP-binding receptors and effectors (*blue*) is the key of the c-di-GMP pleiotropic mechanisms. *B*, spatial localization signals and partner domain occurrence for GGDEF, EAL, and HD-GYP proteins of *P. aeruginosa*. Table based on the work of Seshasayee *et al.* (17) *: The sets of proteins corresponding to each of the category are not mutually exclusive. Organization of classes is in agreement as described previously (17). *TM helices*, transmembrane helices. *C*, pie chart illustrating numerical proportion of GGDEF, EAL, and HD-GYP proteins in *P. aeruginosa*.

The levels of c-di-GMP in the cell are modified by the rate of its synthesis and degradation. The molecule is synthesized from two molecules of GTP by enzymes called diguanylate cyclases (DGCs) and is degraded into 5′-phosphoguanylyl-(3′-5′)-guanosine (pGpG) and/or GMP by phosphodiesterases (PDEs) (Fig. 1*A*). Using bioinformatics, biochemical, and structural approaches, the catalytic domains of DGCs and PDEs have been identified and characterized: the former carrying a GGDEF active site motif, and the latter carrying either EAL or HD-GYP domains (9, 10). These domains can stand alone in a protein or can be present in association with receiver or transmission domains, suggesting a modulation of their enzymatic activity in response to external/internal signals, whereas several have multiple hydrophobic segments, suggesting membrane localization (Fig. 1*B*). This indicates a possible post-translational regulation of DGCs and PDEs that may segregate their activity temporally or spatially. Moreover, GGDEF and EAL domains can both be present in the same protein. In these so-called “hybrid” proteins, either only one of the two domains is catalytically active, the other having acquired a regulatory function, or a third regulatory domain is present, probably disjoining the activity of the GGDEF and EAL domains (11, 12). Recently, examples of proteins with dual DGC and PDE activities have been described, shedding some light on this “biochemical conundrum” (13–15). In *P. aeruginosa*, the GGDEF and the EAL domains of MucR are activated differently so that in planktonic cells, MucR functions as a DGC and as a positive regulator of alginate biosynthesis, whereas in biofilms, it functions as a PDE and is a positive regulator of biofilm dispersal induced by nitric oxide or glutamate (16).

Large-scale genome sequencing led to the discovery that GGDEF- and EAL-containing proteins are nearly ubiquitous in the bacterial kingdom and that bacterial genomes contain multiple copies of genes encoding GGDEF, EAL, or HD-GYP domain-containing proteins (17). A census of all the GGDEF, EAL, and HD-GYP domains in bacterial genomes is available at http://www.ncbi.nlm.nih.gov/Complete_Genomes/c-di-GMP.html (18). The abundance of DGCs and PDEs in a genome may be correlated to the number of complex cellular functions linked with c-di-GMP signaling and to the diversity of possible signals coordinating these functions. The *P. aeruginosa* genome encodes one of the highest numbers of DGCs and PDEs: 18 GGDEF, 5 EAL, 16 GGDEF/EAL, and 3 HD-GYP predicted proteins (supplemental Table S1).

## DGCs: GGDEF Domain Proteins

DGCs function as homodimers. The GGDEF catalytic site is placed at the dimer interface and is involved in the binding of two molecules of GTP and in their conversion into c-di-GMP, with Mg^2+^ as cofactor. Five amino acids upstream of the GGDEF active site is the inhibitory site (I-site) R*XX*D, where the feedback inhibition of the cyclase activity occurs. Binding of c-di-GMP at the I-site prevents the formation of enzymatically active DGC dimers (19). The first experimental demonstration of a DGC activity comes from the work on PleD, a response regulator in *Caulobacter crescentus* (20). Nowadays the PleD activity is well defined together with its receiver (REC) domain and the phosphorylation-induced dimerization. In *P. aeruginosa*, the first biochemical characterization of a DGC stems from the work on WspR, which contains a REC-GGDEF domain organization (supplemental Table S1). The DGC was named after its regulatory role on the *P. aeruginosa* wrinkly spreader phenotype that is correlated with a thick biofilm due to an increased production of exopolysaccharides (21). The control of WspR activity occurs by three different routes that are proposed to occur sub-sequentially. First, upon sensing growth on the surface, the Wsp signal transduction complex phosphorylates WspR and triggers c-di-GMP synthesis (21, 22). In turn, the WspR phosphorylation triggers subcellular WspR oligomerization and cluster formation, which further increases the DGC activity (23). Finally, the feedback inhibition of WspR activity occurs by c-di-GMP binding at the I-site (24). The mechanisms of WspR regulation are supported by structural studies, which revealed that, in solution, the protein can exist in three stable forms: a globular dimer (active), a tetramer (more active), and an elongated dimer (less active due to c-di-GMP binding) (25, 26).

## PDEs: EAL or HD-GYP Domain Proteins

The EAL domain hydrolyzes c-di-GMP into linear pGpG (Fig. 1). Contrary to DGCs, the EAL activity of PDEs seems to be independent of protein oligomerization, whereas it is dependent on binding metal ions (requiring Mg^2+^ or Mn^2+^ and inhibited by Ca^2+^ and Zn^2+^) (27). The glutamate residue (E) in the EAL signature motif is essential, whereas a change of the alanine residue (A) into tyrosine or valine (ETL and EVL) still sustains the enzymatic activity. In *P. aeruginosa*, the CheY-EAL domain protein RocR was identified as a response regulator in the RocSAR signaling system (28). This system is composed of a membrane sensor RocS1 and two response regulators, RocA1 and RocR. RocR activity is triggered by phosphorylation at the CheY domain, and the protein competes with RocA1 for the phosphoryl transfer from the RocS1 sensor. Overall, the Roc system regulates biofilm formation and virulence genes expression (*cup* fimbriae gene clusters and type III secretion system genes) (28, 29).

HD-GYP domain-containing proteins belong to the HD superfamily of metal-dependent phosphohydrolases (11). This enzyme hydrolyzes c-di-GMP in a two-step reaction, producing as a final product two molecules of GMP (Fig. 1). Contrary to GGDEF and EAL proteins, this class of enzyme is not ubiquitous in bacteria, but still widely distributed (18). The first biochemical studies on HD-GYP proteins were conducted on the RpfG PDE from *Xanthomonas campestris* (30). In *P. aeruginosa*, two of the three HD-GYP proteins (PA4108, PA4781, and PA2572) were shown to have a PDE activity *in vivo* and *in vitro* (supplemental Table S1) (31, 32). The structure of PA4781 has been resolved, showing that PA4781 preferentially binds to pGpG over c-di-GMP, and the low rate in hydrolyzing c-di-GMP brought into question its primary work as a genuine PDE (33). Interestingly, pGpG is also a signaling molecule, and it is proposed as a possible alternative to c-di-GMP in certain conditions (3, 31). Finally, the 3′-5′exoribonuclease Orn has been identified in *P. aeruginosa* as primarily responsible for the pGpG cleavage into two GMP molecules (34, 35).

## Discrete Role of DGCs and PDEs on *P. aeruginosa* Biofilm Formation and during Infection

**FIGURE 2.**
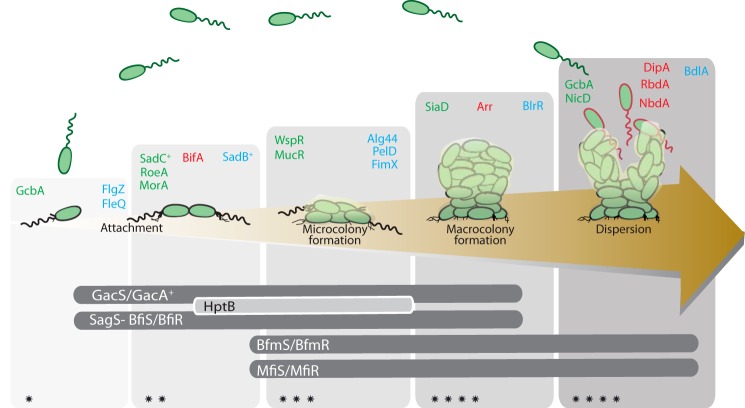
**Coordinated action of c-di-GMP signaling pathways and two-component system cascades in the control of *P. aeruginosa* biofilm development.** In the laboratory, biofilm formation is shown to be a cyclic process that initiates with attachment to the surface of planktonic bacteria (first reversible and then irreversible). A bacteria microcolony is subsequently formed, which evolves into a mature mushroom-shaped macrocolony until the biofilm-associated cells disperse to resume again a planktonic lifestyle. Planktonic, biofilm, and dispersed cells possess distinct physiological stages (*green*, *black*, and *red outline*, respectively) (1, 7). The *upper panel* illustrates DGC (*green*), PDE (*red*), and c-di-GMP receptors/effectors (*blue*) and the developmental stage in which they are proposed to act. Specific references to each DGC/PDE/effector are available in supplemental Tables S1 and S2. The *lower panel* illustrates biofilm stage-specific two-component regulatory systems (45). The gradient of the *gray panels* in the background of the figure indicates increasing intracellular c-di-GMP levels (also indicated with *, **, ***, and ****).

Besides WspR and RocR, described previously, other DGCs and PDEs have been reported as key players in *P. aeruginosa* biofilm formation. Careful examination of *dgc* and *pde* mutant phenotypes, combined with epistasis analysis, pointed at specific features about the role of, for example, SadC and RoeA (DGC) or BifA (PDE) (supplemental Table S1). This resulted in a more global understanding of their relative importance at different stages of the biofilm development process (36, 37). In Fig. 2, we illustrate this concept by including all the *P. aeruginosa* DGCs and PDEs that have been in one way or another associated with biofilm formation. At least five DGCs have been described to specifically control the transition from planktonic to surface-associated growth: WspR, SadC, RoeA, SiaD, and YfiN/TpbB (21, 36–39). Instead, the GcbA and NicD DGCs or the DipA (Pch), RbdA, and NbdA PDEs have been linked to biofilm dispersal (5, 40–44).

**FIGURE 3.**
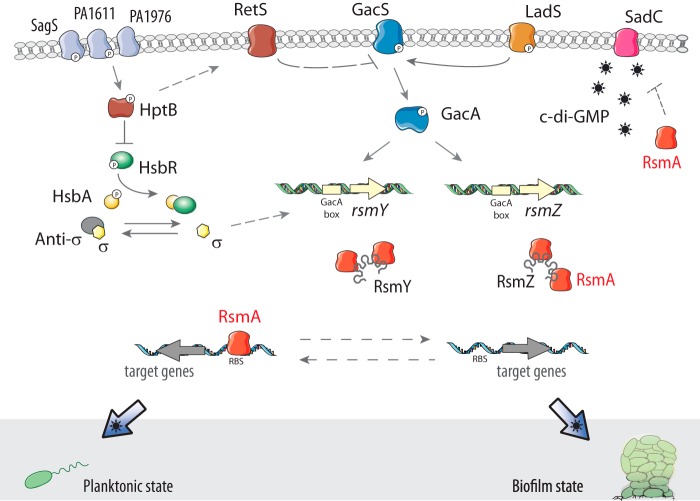
**The Gac/Rsm cascade in *P. aeruginosa* is genetically linked to c-di-GMP through SadC.** The GacS/GacA two-component system is promoting the expression of two small regulatory RNAs, RsmY and RsmZ, which sequester the translational repressor RsmA. Titration of RsmA induces the production of sessile and biofilm determinants, whereas free RsmA leads to a planktonic and more virulent lifestyle (45, 99). Several additional regulators modulate the Gac/Rsm system, such as the two hybrid sensors RetS and LadS, as well as the histidine phosphotransfer protein HptB and other pathways. The elevated concentration of c-di-GMP in a hyperbiofilm-forming *retS* mutant was the first hint of the link between the Gac/Rsm and the c-di-GMP pathways (100). Later on, the molecular details of the link were elucidated: SadC, a DGC whose production is repressed by RsmA, is a central player for the Gac/Rsm regulation of biofilm formation (46). It appears therefore evident that the c-di-GMP signaling network and the Gsc/Rsm cascade are not independent to each other and that they are both instrumental for a proper development of the biofilm.

The sequential intervention of these enzymes reveals that c-di-GMP pathways are well coordinated, organized, insulated, and tuned by global regulatory networks (45). These networks repress or activate distinct c-di-GMP pathways in a defined temporal window. In *P. aeruginosa*, this concept is supported through several examples such as the connection between c-di-GMP signaling and the Gac/Rsm cascade for the control of biofilm formation (Fig. 3), between c-di-GMP signaling and the SagS pathway for the regulation of biofilm antimicrobial resistance, or between c-di-GMP signaling and the Las-mediated quorum-sensing system for the control of biofilm formation and collective motilities (44, 46–48).

*P. aeruginosa* is predominant in chronic infection of cystic fibrosis patients, where the bacterium persists for many years, creating life-threatening lung damage. Over the course of long-term infections, *P. aeruginosa* undergoes extensive genetic and phenotypic adaptation to the lung environment, resulting in a less virulent state with increased production of biofilm (49). A consequence of the *P. aeruginosa* adaptation to the lungs is its phenotypic heterogeneity, *e.g.* the mucoid or the small colony variant (SCV) phenotype (50). In general, SCV colonies appear small, slow growing, and more resistant to several classes of antibiotics, with an increased production of exopolysaccharides and high c-di-GMP levels (50, 51). The c-di-GMP signaling has been proposed to be instrumental for SCV formation because overexpression/activation of DGC such as WspR or YfiN (TbpB) induces the SCV phenotype, whereas mutations in the *wsp* and *yfi* systems were identified in SCVs isolated from cystic fibrosis patients. YfiN is a membrane-anchored DGC, which up-regulates the *pel* and *psl* exopolysaccharide operons (39), whereas its activity is repressed by the YfiR periplasmic protein (52). YfiB is an outer membrane lipoprotein and an antagonist of YfiR (53). Finally, exposure to sub-inhibitory concentration of antibiotic triggers SCVs formation (54, 55), and in the case of kanamycin, this effect is linked to c-di-GMP via the PvrR PDE (55).

## Molecular Mechanisms of c-di-GMP Regulation

The regulation of cellular functions by c-di-GMP occurs at multiple levels, including (i) allosteric regulation of an enzyme activity or protein function, (ii) regulation of gene expression through modulation of a transcription factor, and (iii) regulation of gene expression by direct interaction with noncoding RNA molecules (riboswitches). The molecular bricks by which c-di-GMP builds these regulatory connections are constituted by an array of different c-di-GMP-binding receptors or c-di-GMP effector molecules. We define here c-di-GMP receptors as those molecules that detect c-di-GMP levels in the cell and consequently translate the information into the activation of a specific cellular response/signaling pathway. Instead, c-di-GMP effectors are defined as proteins whose activity changes allosterically upon c-di-GMP binding and consequently regulate a defined interacting target protein. A list of identified c-di-GMP receptors/effectors in *P. aeruginosa* is presented in supplemental Table S2. Among the known c-di-GMP-binding motifs, we include inactive GGDEF, EAL, HD-GYP domains, PilZ domains, and other less characterized examples (11, 56).

In *P. aeruginosa*, PelD is a c-di-GMP receptor whose expression and binding to c-di-GMP are required for Pel polysaccharide production (57). PelD is an inner membrane protein with a GAF domain and a degenerated GGDEF domain with a conserved I-site (supplemental Table S2). The binding of c-di-GMP to PelD occurs at the I-site (57). How the binding stimulates Pel production and/or secretion remains unclear. One can speculate that the c-di-GMP-bound form of PelD interacts with the Pel machinery in a way that induces conformational changes which stimulate exopolysaccharide transport (58, 59).

PilZ domains contain two conserved motifs: an R*XXX*R motif with two conserved arginine residues surrounding one of the c-di-GMP guanine and a D*X*S*XX*G motif that surrounds the other guanine (60). Alg44 is a membrane-associated protein with a cytoplasmic PilZ domain. This protein binds c-di-GMP and is required for *P. aeruginosa* alginate production (61, 62).

Although inactive DGCs, PDEs, and PilZ domains can be recognized *in silico*, other effectors are challenging to identify using bioinformatics prediction. A number of transcriptional regulators have been identified as c-di-GMP receptors. In *P. aeruginosa*, FleQ is an enhancer-binding protein that at low levels of c-di-GMP is the master activator of flagellar gene expression (63). Homologs of FleQ are present in all *Pseudomonas* species and in many flagellated gamma-proteobacteria (64). FleQ does not possess a PilZ domain, but c-di-GMP competitively inhibits FleQ ATPase activity by interacting with the ATP-binding site (65). At high levels of intracellular c-di-GMP, the binding of the molecule to FleQ converts its function as a repressor of the *pel*, *psl*, and *cdr* genes, involved in production of exopolysaccharides and adhesins, into an activator (66). Another c-di-GMP-responsive transcriptional regulator of *P. aeruginosa* is BrlR (67). BrlR participates in the resistance of biofilm cells to antimicrobial agents by increasing the expression of genes encoding multidrug efflux pumps (68, 69). Interestingly, BrlR has a stronger binding affinity for c-di-GMP than FleQ (as characterized by a *K_d_* of 2.2 μm and of 15–20 μm respectively; supplemental Table S2), which suggests that BrlR activation occurs at lower c-di-GMP levels and at earlier stages in the biofilm development process as compared with FleQ (67). In general, determination of the affinity constants of the different receptors or effectors for c-di-GMP can be considered as useful information to determine at which global levels of c-di-GMP they are activated and by extension within which physiological window they act. Finally, c-di-GMP could also act as a competitive inhibitor for certain enzymes capable of catabolizing ATP, such as the FliI flagellar ATPase (70).

The hunt for identifying new c-di-GMP-binding proteins is ongoing, and both *a priori* and *a posteriori* (or targeted) approaches are being employed. *A priori* approaches are based on affinity pulldown assays using c-di-GMP-conjugated Sepharose resin, biotin, or a tripartite c-di-GMP capture compound to enrich c-di-GMP-binding proteins from whole cell lysates (71–73). The differential radial capillary action of ligand assay (DRaCALA) is also used to systematically screen protein expression libraries for their c-di-GMP binding activity(74). Alternatively, the *a posteriori* approaches are “educated guesses,” in which gene products functionally associated with c-di-GMP-regulated processes are tested for c-di-GMP binding via several biochemical assays, among them DRaCALA, isothermal titration calorimetry, and a peptide array approach (74–76).

## The Specificity of c-di-GMP Signaling

A pioneering analysis of all GGDEF and EAL domain-containing proteins from two *P. aeruginosa* strains (PAO1 and PA14), using transposon mutant libraries or strains overexpressing *dgc/pde* genes, revealed that DGCs or PDEs are not redundant and have a different impact on biofilm formation or cytotoxicity (77). Several plausible explanations are proposed for the partial loss or gain of a specific phenotype when deleting a *dgc* or a *pde* gene. One is that DGCs and PDEs are differentially controlled at the level of gene expression or enzyme activity and therefore could have a distinct impact on the global pool of c-di-GMP. Another is related to the degree of c-di-GMP signaling specificity and the existence of local c-di-GMP pools in the cell.

c-di-GMP is a small molecule and presumably diffuses freely in the bacterial cytoplasm. In such a context, all DGCs and PDEs may affect the pool of c-di-GMP uniformly throughout the cell. The degree of c-di-GMP-mediated responses is then possibly determined by the binding affinity of c-di-GMP for different effectors, which in turn leads to various outputs and phenotypes.

The low specificity model does not clash with the idea of a temporal sequestration of DGCs and PDEs. Temporal sequestration is reached by modulation of *dgc* or *pde* gene expression at a defined time period, in response to environmental or cellular alterations through functional association to specific regulatory networks. In *P. aeruginosa*, for example, a case can be made for the repression of SadC by the Gac/Rsm cascade (46), the nutrient-induced activation of the NicD/BdlA/DipA cascade (5), or the presence of Wsp and Yfi multi-protein complexes that control WspR and YfiN DGCs activity, respectively (21, 39, 53).

An alternative hypothesis that may result in highly specific signaling is that each individual DGC and PDE regulates only a subset of c-di-GMP-regulated behaviors. The way this may be achieved is via molecular mechanisms that sequester the signal (c-di-GMP pool) in multi-protein complexes or at distinct cellular sites. An example is the PleD polar sequestration during cell division in *C. crescentus* (20), the YcgR flagellar motor control in *E. coli* and *Salmonella* (78, 79), the PilZ-FimXEAL-c-di-GMP complex of *Xanthomonas citri* (80), the c-di-GMP dependent localization mechanism of LapA in *Pseudomonas fluorescens* (81), or the WspR subcellular clustering in *P. aeruginosa* (23). Interesting lessons on signaling molecule compartmentalization can be taken from cAMP signaling studies in eukaryotes, where the creation of cAMP compartments is achieved mainly by localization of PDEs (82).

It becomes obvious that understanding regulatory mechanisms of DGCs and PDEs is not as simple as measuring global c-di-GMP levels in the cell, and c-di-GMP-dependent control involves highly complex and tightly regulated signaling systems. Low and high signaling specificity could not be mutually exclusive. In the context of c-di-GMP regulation of localized structural machineries, such as flagella or type IV pili, it is reasonable to think that the maintenance of a local c-di-GMP pool would guarantee a more rapid and efficient control of their activity (78–80). Instead, for the overall development of a biofilm, the global c-di-GMP pool may guarantee coordination and cross-talking between multiple pathways (Fig. 2).

## Emerging Challenges in c-di-GMP Signaling Research

Novel and original observations on c-di-GMP signaling in *P. aeruginosa* have recently emerged and have raised new fundamental and challenging questions.

### Heterogeneity of c-di-GMP Levels in Individual Cells

A FRET-based biosensor has been recently constructed and an asymmetrical distribution of c-di-GMP was observed during *P. aeruginosa* and *C. crescentus* cell division (83). The concept of a bimodal distribution of c-di-GMP in *C. crescentus* was not surprising, given its asymmetric cell cycle and the PleD/TipF/PopA localization and activity (8). In the case of *P. aeruginosa*, this observation was more unexpected, as the bacterium produces morphologically similar progeny. Along this line, the same group showed that a specific PDE (named Pch and previously identified as DipA) modulates motility by localizing at the flagellated cell pole. The enzyme is thus asymmetrically partitioned upon cell division to generate c-di-GMP heterogeneity (84). Phenotypic heterogeneity in a population of genetically identical cells has been demonstrated in many bacterial species, particularly for biofilm-forming bacteria. An example is the bistable expression of the biofilm master regulator CsgD in *Salmonella* (85), with CsgD connected to a complex c-di-GMP-dependent regulatory network. Therefore, c-di-GMP might be instrumental for survival and persistence within a changing environment by creating a phenotypic heterogeneous clonal population.

### Cross-talk between Second Messengers

Although c-di-GMP is the second messenger associated with biofilm and chronic infection, cAMP has been shown as being a hallmark for *P. aeruginosa* virulence (*i.e.* acute infection) (86). The dichotomy between these two second messengers is suggested by the observation that increasing c-di-GMP levels, via activation of WspR and YfiN, consequently decreases cAMP levels via an unknown mechanism (87). Interestingly, in the biofilm state, cAMP and c-di-GMP are observed to be spatially organized. Indeed, bacterial cells carrying a cAMP reporter display only little activity in flow chamber-grown biofilm except for cells in the outer layer, whereas a c-di-GMP reporter is overall more active, especially at the bottom of the biofilm and in the middle of microcolonies. Further evidence of a connection between cAMP and c-di-GMP is given by the cAMP-dependent regulation of the minor pilin gene *pilY1*, which seems to activate a signaling cascade causing the increase of c-di-GMP levels during *P. aeruginosa* transition from reversible to irreversible attachment (88). This cross-talk concept is likely to be further expanded and might involve other small molecules such c-di-AMP or ppGpp (89). A *P. aeruginosa* strain lacking (p)ppGpp is sensitive to multiple classes of antibiotics and is defective in biofilm formation (90). The connection between c-di-GMP and (p)ppGpp has been recently proposed in *Mycobacterium smegmatis*, where both signaling molecules may be involved in the metabolism of glycopeptidolipids and polar lipids, leading to an increase of the bacterium antibiotic resistance (91).

### c-di-GMP Regulation of Antimicrobial Resistance

Cells in a biofilm can be up to 1000 times less susceptible to antimicrobial agents than planktonic cells (92). The reasons for the biofilm tolerance are multiple, including slow growth or the presence of an extracellular matrix (93, 94). By regulating biofilm, c-di-GMP signaling can therefore also influence the antimicrobial resistance of the bacterium. Recently, new c-di-GMP-related mechanisms have been described to contribute to *P. aeruginosa* antibiotic resistance, independently from biofilm formation. A *pel* mutant strain with high c-di-GMP levels (overexpression of the PA5487 DGC) has a higher fitness in the presence of imipenem as compared with the same strain with low c-di-GMP levels (PvrR PDE overexpression) (95). Sub-inhibitory concentrations of aminoglycosides induce biofilm formation in terms of biomass but are not linked to exopolysaccharide production. The PDE Arr has been demonstrated to be necessary for such a response (96). Finally, lowering c-di-GMP levels in *P. aeruginosa* by engineering a *sagS* deletion renders the bacterium more susceptible to antibiotics, whereas this strain is still capable of forming proper biofilms (47, 48). Furthermore, upon overexpression of the AdcA DGC, resistance to antibiotics is restored to wild type levels (48).

Overall, the possibility to fight against biofilm formation, antimicrobial resistance, and chronic infections by manipulating and subverting c-di-GMP signaling is an interesting therapeutic challenge (97, 98). The targets are multiple and give the opportunity to intervene at a global level by targeting DGCs or PDEs, or to be more clinical by aiming at specific receptors/effectors and thus inhibit specific pathways.

## Final Remarks and Future Perspectives

*P. aeruginosa* has come to be a remarkable model organism for bacterial pathogenesis (2, 55, 93). Nowadays a wide variety of technical tools are available for researchers who intend to study this microorganism. The significant progresses that have been made in understanding c-di-GMP-regulated phenotypes in *P. aeruginosa* could therefore be applicable to other bacteria that are relatively less easy to manipulate in the laboratory.

Importantly, despite this progress, many questions about c-di-GMP mechanisms of action remain unanswered. The basics of c-di-GMP metabolism have been elucidated, and we understand most of the enzymology behind its synthesis and degradation. However, the detailed mechanisms through which c-di-GMP operates, and in particular the process of specific transmission, remain obscure. Identification of new c-di-GMP receptors/effectors surely helps researchers in making better connections between c-di-GMP signaling and functional output. Now, have we identified all the players and their role in the c-di-GMP contest? Surely not! In the case of the c-di-GMP regulation of exopolysaccharide production/secretion in *P. aeruginosa*, for example, although a good number of involved DGCs/PDEs/effectors have been identified, *e.g.* PelD or Alg44, how they act on the associated molecular mechanism(s) remains to be deciphered.

## Supplementary Material

Supplemental Data

## Footnotes

3 The abbreviations used are:

c-di-GMP: cyclic-di-GMP
DGC: diguanylate cyclase
PDE: phosphodiesterase
I-site: inhibitory site
SCV: small colony variant
guanosine pGpG: 5′-phosphoguanylyl-(3′-5′)-guanosine
ppGpp: guanosine tetraphosphate
(p)ppGpp: tetra- or pentaphosphate guanosine.
