# Supplementary material for: Biofilms and Cyclic di-GMP (c-di-GMP) Signaling: Lessons from Pseudomonas aeruginosa and Other Bacteria
Source: J Biol Chem. 2016 Apr 21;291(24):12547–55. doi: 10.1074/jbc.R115.711507 (PMC4933438; doi:10.1074/jbc.R115.711507)
Supplement: Supplemental Data [file supp_291_24_12547__index.html]

Biofilms and c-di-GMP Signaling: Lessons from Pseudomonas aeruginosa and other Bacteria — Biofilms and Cyclic di-GMP (c-di-GMP) Signaling: Lessons from Pseudomonas aeruginosa and Other Bacteria — MINIREVIEW: Biofilm and c-di-GMP Signaling — Supplemental Data 

# Biofilms and Cyclic di-GMP (c-di-GMP) Signaling: Lessons from *Pseudomonas aeruginosa* and Other Bacteria

## Supplemental Data

- Supplemental tables (.docx, 189 KB) - Supplemental tables 1 and 2 together with associated references
