# Supplementary material for: Biofilms and Cyclic di-GMP (c-di-GMP) Signaling: Lessons from Pseudomonas aeruginosa and Other Bacteria
Source: J Biol Chem. 2016 Apr 21;291(24):12547–55. doi: 10.1074/jbc.R115.711507 (PMC4933438; doi:10.1074/jbc.R115.711507)
Supplement: Supplemental Data [file 10.1074_R115.711507_jbc.R115.711507-1.docx]

**Table S1: List of all GGDEF, EAL, HD-GYP proteins in *P. aeruginosa*.**

| **ORF** | **Name** | **Domain signature^#^** | **i-site^#^** | **Additional domains^#^** | **Activity^+^** | **Function** | **References** |
| --- | --- | --- | --- | --- | --- | --- | --- |
| PA0169 | *siaD* | GGEEF | Y |  | DGC | Autoaggregation, EPS production | ([1-4](#_ENREF_1)) |
| PA0285 |  | GGDEF, ESL | N | TM, PAS | ND |  |  |
| PA0290 |  | GGEEF | Y | PAS | ND |  |  |
| PA0338 |  | GGEEF | Y | PAS | ND |  |  |
| PA0575 |  | GGDEF, EAL | Y | PBPb, TM, PAS | ND |  |  |
| PA0707 | *toxR, regA* | EAL | NA |  | ND | Regulator of exotoxin A | ([5](#_ENREF_5)) |
| PA0847 |  | GGDEF | Y | TM, Chase4, HAMP, PAS | DGC |  |  |
| PA0861 | *rbdA* | GGDEF, ELL | Y | TM, PAS | PDE | Biofilm dispersal | ([6](#_ENREF_6)) |
| PA1107 | *roeA* | GGEEF | Y | TM | DGC | EPS production | ([7](#_ENREF_7),[8](#_ENREF_8)) |
| PA1120 | *yfiN, tpbB* | GGDEF | Y | HAMP | DGC | Small colony variants appearance | ([9-14](#_ENREF_9)) |
| PA1181 |  | GGDEF, ELL | Y | MASE, PAS | ND |  |  |
| PA1433 | ***lapD**** | RGGEF, KVL | N | HAMP | - | c-di-GMP receptor, surface attachment | ([15-18](#_ENREF_15)) |
| PA1727 | *mucR* | GGDEF, EAL | Y | MHYT | DGC, PDE | Alginate production, biofilm dispersal | ([19-21](#_ENREF_19)) |
| PA1851 |  | GGEEF | Y | TM | ND |  |  |
| PA2072 |  | GGDEF, EAL | Y | Chase4, TM, PAS | PDE |  |  |
| PA2133 |  | ETL | NA |  | PDE |  |  |
| PA2200 |  | EAL | NA | TM | PDE |  |  |
| PA2567 |  | SPTFR, EAL | N | GAF | PDE |  |  |
| PA2572 |  | YN-GYP | NA | Rec | - | Biofilm formation | ([22](#_ENREF_22)) |
| PA2771 |  | GGEEF | Y | GAF | ND |  |  |
| PA2818 | *arr** | EAL | NA | TM | PDE | Aminoglycoside response regulator | ([23](#_ENREF_23),[24](#_ENREF_24)) |
| PA2870 |  | GGEEF | Y | TM | DGC |  |  |
| PA3061 | ***pelD*** | RNDEG | Y | TM, GAF | - | c-di-GMP receptor involved in pellicle formation | ([25-27](#_ENREF_25)) |
| PA3177 |  | GGEEF | Y |  | ND |  |  |
| PA3258 |  | EAL, GGDDF | N | CBS | ND |  |  |
| PA3311 | *nbdA* | AGDEF, EAL | Y | TM, MHYT | PDE | Biofilm dispersal | ([20](#_ENREF_20),[28](#_ENREF_28)) |
| PA3343 |  | GGEEF | Y | TM | DGC |  |  |
| PA3702 | *wspR* | GGEEF | Y | Rec | DGC | Biofilm formation | ([29-34](#_ENREF_29)) |
| PA3825 |  | EVL | NA |  | PDE |  |  |
| PA3947 | *rocR* | EVL | NA | Rec | PDE | Biofilm formation, virulence | ([35-40](#_ENREF_35)) |
| PA4108 |  | HD-GYP | NA |  | PDE |  | ([22](#_ENREF_22)) |
| PA4332 | *sadC* | GGEEF | Y | TM | DGC | Biofilm formation, flagellar motility | ([3](#_ENREF_3),[8](#_ENREF_8),[41](#_ENREF_41),[42](#_ENREF_42)) |
| PA4367 | *bifA* | GGDQF, EAL | N | TM | PDE | Biofilm formation, swarming motility | ([41](#_ENREF_41),[43](#_ENREF_43)) |
| PA4396 |  | DEQFH | Y | Rec | DGC |  |  |
| PA4601 | *morA* | GGDEF, EAL | N | TM, PAS | ND | Virulence, Type II secretion | ([44-46](#_ENREF_44)) |
| PA4781 |  | HD-GYP | NA | Rec | PDE | Biofilm formation | ([22](#_ENREF_22),[47](#_ENREF_47),[48](#_ENREF_48)) |
| PA4843 | *acbA* | GGEEF | Y | Rec | DGC | Biofilm development | ([49](#_ENREF_49)) |
| PA4929 | *nicD* | GGDEF | Y | 7TMR_DISM | DGC | Nutrient-induced biofilm dispersal | ([28](#_ENREF_28)) |
| PA4959 | ***fimX*** | GDSIF, EVL | N | PAS | - | c-di-GMP receptor, twitching motility, biofilm formation | ([50-54](#_ENREF_50)) |
| PA5017 | *dipA, pch* | ASNEF, EAL | N | PAS, GAF | PDE | Biofilm dispersal, c-di-GMP heterogeneity | ([55](#_ENREF_55),[56](#_ENREF_56)) |
| PA5295 |  | GSDEF, EAL | N |  | ND |  |  |
| PA5442 |  | AGDEF, EAL | N | TM, PAS | ND |  |  |
| PA5487 | *dgcP** | GGEEF | Y |  | DGC | Biofilm formation | ([57](#_ENREF_57)) |
| PA14_59790 | *pvrR** | EAL | NA | Rec | PDE | Biofilm formation | ([58](#_ENREF_58)) |

#: as illustrated here ([59](#_ENREF_59)). Y:yes; N:no; N/A: not applicable; ND:not determined; -:not active +: where not indicated by specific paper, the DGC/PDE activity was detected in a high-throughput screening ([60](#_ENREF_60)). *: based on homologous protein found in other *Pseudomonas* species or, in case of PvrR, only in *P. aeruginosa* PA14 and PA7.

**Table S2: List of identified c-di-GMP receptors/effectors in *P. aeruginosa*.**

| **ORF** | **Name** | **Domain**  **signature^#^** | **Additional domains^#^** | **Binding** | **Role** | **PBD** | **References** |
| --- | --- | --- | --- | --- | --- | --- | --- |
| PA0012 |  | PilZ |  | 402nM | Unknown |  | ([61](#_ENREF_61)) |
| PA2799 |  | PilZ |  | 2µM | Unknown |  | ([61](#_ENREF_61)) |
| PA2960 | *pilZ* | PilZ |  | - | Twitching motility |  | ([62](#_ENREF_62)) |
| PA2989 |  | PilZ |  | 288nM | Unknown |  | ([61](#_ENREF_61)) |
| PA3061 | *pelD* | RNDEG | TM, GAF | 1-2 µM | Pellicle formation | 4ETX/V/Z, 4EU0, 4DMZ, 4DN0 | ([25-27](#_ENREF_25)) |
| PA3353 | *flgZ* | YgcR-like |  | Unknown | Flagellar motility |  | ([63](#_ENREF_63)) |
| PA3542 | *alg44* | PilZ | TM | 8.4µM | Alginate biosynthesis |  | ([61](#_ENREF_61),[64](#_ENREF_64),[65](#_ENREF_65)) |
| PA3702 | *fleQ* | Uknown | Rec, AAA+ ATPase, HTH DNA binding | 15-20 µM | Transcriptional regulator, EPS and flagellar gene expression | 4WXM | ([66](#_ENREF_66),[67](#_ENREF_67)) |
| PA3740 |  | LKKALKKQTNLR |  | 30 nM | Swimming, swarming, growth (lag phase) |  | ([68](#_ENREF_68)) |
| PA4324 |  | PilZ |  | Unknown | Unknown |  |  |
| PA4608 |  | PilZ |  | 6.1µM | Unknown | 1YWU | ([61](#_ENREF_61),[69](#_ENREF_69)) |
| PA4878 | *brlR* | Unknown | HTH DNA binding, GyrI-like binding | 2.2 µM | Biofilm resistance |  | ([70](#_ENREF_70)) |
| PA4959 | *fimX* | GDSIF, EVL | PAS | 125 nM | Twitching motility, biofilm formation | 3HV8/9, 3HVA/B | ([50-54](#_ENREF_50)) |

#: as illustrated here ([59](#_ENREF_59)).

**Supplemental References**

1. Chen, Y., Yuan, M., Mohanty, A., Yam, J. K., Liu, Y., Chua, S. L., Nielsen, T. E., Tolker-Nielsen, T., Givskov, M., Cao, B., and Yang, L. (2015) Multiple diguanylate cyclase-coordinated regulation of pyoverdine synthesis in Pseudomonas aeruginosa. *Environ Microbiol Rep* **7**, 498-507

2. Chua, S. L., Sivakumar, K., Rybtke, M., Yuan, M., Andersen, J. B., Nielsen, T. E., Givskov, M., Tolker-Nielsen, T., Cao, B., Kjelleberg, S., and Yang, L. (2015) C-di-GMP regulates Pseudomonas aeruginosa stress response to tellurite during both planktonic and biofilm modes of growth. *Sci Rep* **5**, 10052

3. Irie, Y., Borlee, B. R., O'Connor, J. R., Hill, P. J., Harwood, C. S., Wozniak, D. J., and Parsek, M. R. (2012) Self-produced exopolysaccharide is a signal that stimulates biofilm formation in Pseudomonas aeruginosa. *Proc Natl Acad Sci U S A* **109**, 20632-20636

4. Klebensberger, J., Birkenmaier, A., Geffers, R., Kjelleberg, S., and Philipp, B. (2009) SiaA and SiaD are essential for inducing autoaggregation as a specific response to detergent stress in *Pseudomonas aeruginosa*. *Environ Microbiol* **11**, 3073-3086

5. Walker, S. L., Hiremath, L. S., Wozniak, D. J., and Galloway, D. R. (1994) ToxR (RegA)-mediated in vitro transcription of Pseudomonas aeruginosa toxA. *Gene* **150**, 87-92

6. An, S., Wu, J., and Zhang, L. H. (2010) Modulation of *Pseudomonas aeruginosa* biofilm dispersal by a cyclic-Di-GMP phosphodiesterase with a putative hypoxia-sensing domain. *Appl Environ Microbiol* **76**, 8160-8173

7. Bernier, S. P., Ha, D. G., Khan, W., Merritt, J. H., and O'Toole, G. A. (2011) Modulation of *Pseudomonas aeruginosa* surface-associated group behaviors by individual amino acids through c-di-GMP signaling. *Res Microbiol* **162**, 680-688

8. Merritt, J. H., Ha, D. G., Cowles, K. N., Lu, W., Morales, D. K., Rabinowitz, J., Gitai, Z., and O'Toole, G. A. (2010) Specific control of *Pseudomonas aeruginosa* surface-associated behaviors by two c-di-GMP diguanylate cyclases. *MBio* **1**

9. Malone, J. G., Jaeger, T., Manfredi, P., Dotsch, A., Blanka, A., Bos, R., Cornelis, G. R., Haussler, S., and Jenal, U. (2012) The YfiBNR signal transduction mechanism reveals novel targets for the evolution of persistent *Pseudomonas aeruginosa* in cystic fibrosis airways. *PLoS Pathog* **8**, e1002760

10. Malone, J. G., Jaeger, T., Spangler, C., Ritz, D., Spang, A., Arrieumerlou, C., Kaever, V., Landmann, R., and Jenal, U. (2010) YfiBNR mediates cyclic di-GMP dependent small colony variant formation and persistence in *Pseudomonas aeruginosa*. *PLoS Pathog* **6**, e1000804

11. Giardina, G., Paiardini, A., Fernicola, S., Franceschini, S., Rinaldo, S., Stelitano, V., and Cutruzzola, F. (2013) Investigating the allosteric regulation of YfiN from Pseudomonas aeruginosa: clues from the structure of the catalytic domain. *PLoS One* **8**, e81324

12. Li, S., Li, T., Xu, Y., Zhang, Q., Zhang, W., Che, S., Liu, R., Wang, Y., and Bartlam, M. (2015) Structural insights into YfiR sequestering by YfiB in *Pseudomonas aeruginosa* PAO1. *Sci Rep* **5**, 16915

13. Xu, K., Li, S., Yang, W., Li, K., Bai, Y., Xu, Y., Jin, J., Wang, Y., and Bartlam, M. (2015) Structural and Biochemical Analysis of Tyrosine Phosphatase Related to Biofilm Formation A (TpbA) from the Opportunistic Pathogen Pseudomonas aeruginosa PAO1. *PLoS One* **10**, e0124330

14. Yang, X., Yang, X. A., Xu, M., Zhou, L., Fan, Z., and Jiang, T. (2015) Crystal structures of YfiR from Pseudomonas aeruginosa in two redox states. *Biochem Biophys Res Commun* **461**, 14-20

15. Hinsa, S. M., and O'Toole, G. A. (2006) Biofilm formation by Pseudomonas fluorescens WCS365: a role for LapD. *Microbiology* **152**, 1375-1383

16. Navarro, M. V., Newell, P. D., Krasteva, P. V., Chatterjee, D., Madden, D. R., O'Toole, G. A., and Sondermann, H. (2011) Structural basis for c-di-GMP-mediated inside-out signaling controlling periplasmic proteolysis. *PLoS Biol* **9**, e1000588

17. Newell, P. D., Boyd, C. D., Sondermann, H., and O'Toole, G. A. (2011) A c-di-GMP effector system controls cell adhesion by inside-out signaling and surface protein cleavage. *PLoS Biol* **9**, e1000587

18. Newell, P. D., Monds, R. D., and O'Toole, G. A. (2009) LapD is a bis-(3',5')-cyclic dimeric GMP-binding protein that regulates surface attachment by Pseudomonas fluorescens Pf0-1. *Proc Natl Acad Sci U S A* **106**, 3461-3466

19. Hay, I. D., Remminghorst, U., and Rehm, B. H. (2009) MucR, a novel membrane-associated regulator of alginate biosynthesis in *Pseudomonas aeruginosa*. *Appl Environ Microbiol* **75**, 1110-1120

20. Li, Y., Heine, S., Entian, M., Sauer, K., and Frankenberg-Dinkel, N. (2013) NO-induced biofilm dispersion in *Pseudomonas aeruginosa* is mediated by an MHYT domain-coupled phosphodiesterase. *J Bacteriol* **195**, 3531-3542

21. Wang, Y., Hay, I. D., Rehman, Z. U., and Rehm, B. H. (2015) Membrane-anchored MucR mediates nitrate-dependent regulation of alginate production in *Pseudomonas aeruginosa*. *Appl Microbiol Biotechnol* **99**, 7253-7265

22. Ryan, R. P., Lucey, J., O'Donovan, K., McCarthy, Y., Yang, L., Tolker-Nielsen, T., and Dow, J. M. (2009) HD-GYP domain proteins regulate biofilm formation and virulence in *Pseudomonas aeruginosa*. *Environ Microbiol* **11**, 1126-1136

23. Hoffman, L. R., D'Argenio, D. A., MacCoss, M. J., Zhang, Z., Jones, R. A., and Miller, S. I. (2005) Aminoglycoside antibiotics induce bacterial biofilm formation. *Nature* **436**, 1171-1175

24. Gotoh, H., Zhang, Y., Dallo, S. F., Hong, S., Kasaraneni, N., and Weitao, T. (2008) Pseudomonas aeruginosa, under DNA replication inhibition, tends to form biofilms via Arr. *Res Microbiol* **159**, 294-302

25. Lee, V. T., Matewish, J. M., Kessler, J. L., Hyodo, M., Hayakawa, Y., and Lory, S. (2007) A cyclic-di-GMP receptor required for bacterial exopolysaccharide production. *Mol Microbiol* **65**, 1474-1484

26. Li, Z., Chen, J. H., Hao, Y., and Nair, S. K. (2012) Structures of the PelD cyclic diguanylate effector involved in pellicle formation in *Pseudomonas aeruginosa* PAO1. *J Biol Chem* **287**, 30191-30204

27. Whitney, J. C., Colvin, K. M., Marmont, L. S., Robinson, H., Parsek, M. R., and Howell, P. L. (2012) Structure of the cytoplasmic region of PelD, a degenerate diguanylate cyclase receptor that regulates exopolysaccharide production in *Pseudomonas aeruginosa*. *J Biol Chem* **287**, 23582-23593

28. Basu Roy, A., and Sauer, K. (2014) Diguanylate cyclase NicD-based signalling mechanism of nutrient-induced dispersion by *Pseudomonas aeruginosa*. *Mol Microbiol* **94**, 771-793

29. De, N., Navarro, M. V., Wang, Q., Krasteva, P. V., and Sondermann, H. (2010) Biophysical assays for protein interactions in the Wsp sensory system and biofilm formation. *Methods Enzymol* **471**, 161-184

30. De, N., Pirruccello, M., Krasteva, P. V., Bae, N., Raghavan, R. V., and Sondermann, H. (2008) Phosphorylation-independent regulation of the diguanylate cyclase WspR. *PLoS Biol* **6**, e67

31. Guvener, Z. T., and Harwood, C. S. (2007) Subcellular location characteristics of the *Pseudomonas aeruginosa* GGDEF protein, WspR, indicate that it produces cyclic-di-GMP in response to growth on surfaces. *Mol Microbiol* **66**, 1459-1473

32. Hickman, J. W., Tifrea, D. F., and Harwood, C. S. (2005) A chemosensory system that regulates biofilm formation through modulation of cyclic diguanylate levels. *Proc Natl Acad Sci U S A* **102**, 14422-14427

33. Huangyutitham, V., Guvener, Z. T., and Harwood, C. S. (2013) Subcellular clustering of the phosphorylated WspR response regulator protein stimulates its diguanylate cyclase activity. *MBio* **4**, e00242-00213

34. Malone, J. G., Williams, R., Christen, M., Jenal, U., Spiers, A. J., and Rainey, P. B. (2007) The structure-function relationship of WspR, a *Pseudomonas fluorescens* response regulator with a GGDEF output domain. *Microbiology* **153**, 980-994

35. Chen, M. W., Kotaka, M., Vonrhein, C., Bricogne, G., Rao, F., Chuah, M. L., Svergun, D., Schneider, G., Liang, Z. X., and Lescar, J. (2012) Structural insights into the regulatory mechanism of the response regulator RocR from *Pseudomonas aeruginosa* in cyclic Di-GMP signaling. *J Bacteriol* **194**, 4837-4846

36. Cohen, D., Mechold, U., Nevenzal, H., Yarmiyhu, Y., Randall, T. E., Bay, D. C., Rich, J. D., Parsek, M. R., Kaever, V., Harrison, J. J., and Banin, E. (2015) Oligoribonuclease is a central feature of cyclic diguanylate signaling in *Pseudomonas aeruginosa*. *Proc Natl Acad Sci U S A* **112**, 11359-11364

37. Kotaka, M., Dutta, S., Lee, H. C., Lim, M. J., Wong, Y., Rao, F., Mitchell, E. P., Liang, Z. X., and Lescar, J. (2009) Expression, purification and preliminary crystallographic analysis of Pseudomonas aeruginosa RocR protein. *Acta Crystallogr Sect F Struct Biol Cryst Commun* **65**, 1035-1038

38. Kulasekara, H. D., Ventre, I., Kulasekara, B. R., Lazdunski, A., Filloux, A., and Lory, S. (2005) A novel two-component system controls the expression of *Pseudomonas aeruginosa* fimbrial cup genes. *Mol Microbiol* **55**, 368-380

39. Rao, F., Qi, Y., Chong, H. S., Kotaka, M., Li, B., Li, J., Lescar, J., Tang, K., and Liang, Z. X. (2009) The functional role of a conserved loop in EAL domain-based cyclic di-GMP-specific phosphodiesterase. *J Bacteriol* **191**, 4722-4731

40. Rao, F., Yang, Y., Qi, Y., and Liang, Z. X. (2008) Catalytic mechanism of cyclic di-GMP-specific phosphodiesterase: a study of the EAL domain-containing RocR from *Pseudomonas aeruginosa*. *J Bacteriol* **190**, 3622-3631

41. Merritt, J. H., Brothers, K. M., Kuchma, S. L., and O'Toole, G. A. (2007) SadC reciprocally influences biofilm formation and swarming motility via modulation of exopolysaccharide production and flagellar function. *J Bacteriol* **189**, 8154-8164

42. Moscoso, J. A., Jaeger, T., Valentini, M., Hui, K., Jenal, U., and Filloux, A. (2014) The diguanylate cyclase SadC is a central player in Gac/Rsm-mediated biofilm formation in *Pseudomonas aeruginosa*. *J Bacteriol* **196**, 4081-4088

43. Kuchma, S. L., Brothers, K. M., Merritt, J. H., Liberati, N. T., Ausubel, F. M., and O'Toole, G. A. (2007) BifA, a cyclic-Di-GMP phosphodiesterase, inversely regulates biofilm formation and swarming motility by Pseudomonas aeruginosa PA14. *J Bacteriol* **189**, 8165-8178

44. Choy, W. K., Zhou, L., Syn, C. K., Zhang, L. H., and Swarup, S. (2004) MorA defines a new class of regulators affecting flagellar development and biofilm formation in diverse Pseudomonas species. *J Bacteriol* **186**, 7221-7228

45. Phippen, C. W., Mikolajek, H., Schlaefli, H. G., Keevil, C. W., Webb, J. S., and Tews, I. (2014) Formation and dimerization of the phosphodiesterase active site of the Pseudomonas aeruginosa MorA, a bi-functional c-di-GMP regulator. *FEBS Lett* **588**, 4631-4636

46. Ravichandran, A., Ramachandran, M., Suriyanarayanan, T., Wong, C. C., and Swarup, S. (2015) Global Regulator MorA Affects Virulence-Associated Protease Secretion in Pseudomonas aeruginosa PAO1. *PLoS One* **10**, e0123805

47. Rinaldo, S., Paiardini, A., Stelitano, V., Brunotti, P., Cervoni, L., Fernicola, S., Protano, C., Vitali, M., Cutruzzola, F., and Giardina, G. (2015) Structural basis of functional diversification of the HD-GYP domain revealed by the *Pseudomonas aeruginosa* PA4781 protein, which displays an unselective bimetallic binding site. *J Bacteriol* **197**, 1525-1535

48. Stelitano, V., Giardina, G., Paiardini, A., Castiglione, N., Cutruzzola, F., and Rinaldo, S. (2013) C-di-GMP hydrolysis by *Pseudomonas aeruginosa* HD-GYP phosphodiesterases: analysis of the reaction mechanism and novel roles for pGpG. *PLoS One* **8**, e74920

49. Jones, C. J., Newsom, D., Kelly, B., Irie, Y., Jennings, L. K., Xu, B., Limoli, D. H., Harrison, J. J., Parsek, M. R., White, P., and Wozniak, D. J. (2014) ChIP-Seq and RNA-Seq reveal an AmrZ-mediated mechanism for cyclic di-GMP synthesis and biofilm development by Pseudomonas aeruginosa. *PLoS Pathog* **10**, e1003984

50. Kazmierczak, B. I., Lebron, M. B., and Murray, T. S. (2006) Analysis of FimX, a phosphodiesterase that governs twitching motility in Pseudomonas aeruginosa. *Mol Microbiol* **60**, 1026-1043

51. Huang, B., Whitchurch, C. B., and Mattick, J. S. (2003) FimX, a multidomain protein connecting environmental signals to twitching motility in Pseudomonas aeruginosa. *J Bacteriol* **185**, 7068-7076

52. Jain, R., Behrens, A. J., Kaever, V., and Kazmierczak, B. I. (2012) Type IV pilus assembly in Pseudomonas aeruginosa over a broad range of cyclic di-GMP concentrations. *J Bacteriol* **194**, 4285-4294

53. Navarro, M. V., De, N., Bae, N., Wang, Q., and Sondermann, H. (2009) Structural analysis of the GGDEF-EAL domain-containing c-di-GMP receptor FimX. *Structure* **17**, 1104-1116

54. Qi, Y., Xu, L., Dong, X., Yau, Y. H., Ho, C. L., Koh, S. L., Shochat, S. G., Chou, S. H., Tang, K., and Liang, Z. X. (2012) Functional divergence of FimX in PilZ binding and type IV pilus regulation. *J Bacteriol* **194**, 5922-5931

55. Li, Y., Petrova, O. E., Su, S., Lau, G. W., Panmanee, W., Na, R., Hassett, D. J., Davies, D. G., and Sauer, K. (2014) BdlA, DipA and induced dispersion contribute to acute virulence and chronic persistence of Pseudomonas aeruginosa. *PLoS Pathog* **10**, e1004168

56. Roy, A. B., Petrova, O. E., and Sauer, K. (2012) The phosphodiesterase DipA (PA5017) is essential for *Pseudomonas aeruginosa* biofilm dispersion. *J Bacteriol* **194**, 2904-2915

57. Aragon, I. M., Perez-Mendoza, D., Moscoso, J. A., Faure, E., Guery, B., Gallegos, M. T., Filloux, A., and Ramos, C. (2015) Diguanylate cyclase DgcP is involved in plant and human Pseudomonas spp. infections. *Environ Microbiol*

58. Mikkelsen, H., Ball, G., Giraud, C., and Filloux, A. (2009) Expression of Pseudomonas aeruginosa CupD fimbrial genes is antagonistically controlled by RcsB and the EAL-containing PvrR response regulators. *PLoS One* **4**, e6018

59. Christen, M., Christen, B., Folcher, M., Schauerte, A., and Jenal, U. (2005) Identification and characterization of a cyclic di-GMP-specific phosphodiesterase and its allosteric control by GTP. *J Biol Chem* **280**, 30829-30837

60. Kulasakara, H., Lee, V., Brencic, A., Liberati, N., Urbach, J., Miyata, S., Lee, D. G., Neely, A. N., Hyodo, M., Hayakawa, Y., Ausubel, F. M., and Lory, S. (2006) Analysis of *Pseudomonas aeruginosa* diguanylate cyclases and phosphodiesterases reveals a role for bis-(3'-5')-cyclic-GMP in virulence. *Proc Natl Acad Sci U S A* **103**, 2839-2844

61. Christen, M., Kulasekara, H. D., Christen, B., Kulasekara, B. R., Hoffman, L. R., and Miller, S. I. (2010) Asymmetrical distribution of the second messenger c-di-GMP upon bacterial cell division. *Science* **328**, 1295-1297

62. Alm, R. A., Bodero, A. J., Free, P. D., and Mattick, J. S. (1996) Identification of a novel gene, pilZ, essential for type 4 fimbrial biogenesis in Pseudomonas aeruginosa. *J Bacteriol* **178**, 46-53

63. Martinez-Granero, F., Navazo, A., Barahona, E., Redondo-Nieto, M., Gonzalez de Heredia, E., Baena, I., Martin-Martin, I., Rivilla, R., and Martin, M. (2014) Identification of flgZ as a flagellar gene encoding a PilZ domain protein that regulates swimming motility and biofilm formation in Pseudomonas. *PLoS One* **9**, e87608

64. Remminghorst, U., and Rehm, B. H. (2006) Alg44, a unique protein required for alginate biosynthesis in Pseudomonas aeruginosa. *FEBS Lett* **580**, 3883-3888

65. Merighi, M., Lee, V. T., Hyodo, M., Hayakawa, Y., and Lory, S. (2007) The second messenger bis-(3'-5')-cyclic-GMP and its PilZ domain-containing receptor Alg44 are required for alginate biosynthesis in *Pseudomonas aeruginosa*. *Mol Microbiol* **65**, 876-895

66. Hickman, J. W., and Harwood, C. S. (2008) Identification of FleQ from *Pseudomonas aeruginosa* as a c-di-GMP-responsive transcription factor. *Mol Microbiol* **69**, 376-389

67. Su, T., Liu, S., Wang, K., Chi, K., Zhu, D., Wei, T., Huang, Y., Guo, L., Hu, W., Xu, S., Lin, Z., and Gu, L. (2015) The REC domain mediated dimerization is critical for FleQ from *Pseudomonas aeruginosa* to function as a c-di-GMP receptor and flagella gene regulator. *J Struct Biol* **192**, 1-13

68. Duvel, J., Bense, S., Moller, S., Bertinetti, D., Schwede, F., Morr, M., Eckweiler, D., Genieser, H. G., Jansch, L., Herberg, F. W., Frank, R., and Haussler, S. (2015) Application of synthetic peptide arrays to uncover c-di-GMP binding motifs. *J Bacteriol*

69. Habazettl, J., Allan, M. G., Jenal, U., and Grzesiek, S. (2011) Solution structure of the PilZ domain protein PA4608 complex with cyclic di-GMP identifies charge clustering as molecular readout. *J Biol Chem* **286**, 14304-14314

70. Chambers, J. R., Liao, J., Schurr, M. J., and Sauer, K. (2014) BrlR from *Pseudomonas aeruginosa* is a c-di-GMP-responsive transcription factor. *Mol Microbiol* **92**, 471-487
